# Genesis and mechanisms controlling tornillo seismo-volcanic events in volcanic areas

**DOI:** 10.1038/s41598-019-43842-y

**Published:** 2019-05-14

**Authors:** Marco Fazio, Salvatore Alparone, Philip M. Benson, Andrea Cannata, Sergio Vinciguerra

**Affiliations:** 1 Rock Mechanics Laboratory, University of Portsmouth, Portsmouth, PO1 3QL UK; 2 Department of Applied Geology, University of Göttingen, Göttingen, 37077 Germany; 3 Istituto Nazionale di Geofisica e Vulcanologia, Osservatorio Etneo - Sezione di Catania, 95125 Catania, Italy; 4 Department of Physics and Geology, University of Perugia, Perugia, 06123 Italy; 5 Department of Earth Sciences, University of Turin, Turin, 10125 Italy; 6 Present Address: Dipartimento di Scienze Biologiche, Geologiche e Ambientali – Sezione di Scienze della Terra, Universita’ degli Studi di Catania, 95129 Catania, Italy

**Keywords:** Seismology, Volcanology

## Abstract

Volcanic activity is often preceded or accompanied by different types of seismo-volcanic signals. Among these signals, the so-called tornillo (Spanish for “screw”) events are considered to belong to a unique class of volcano-seismicity characterised by a long-duration coda, amplitude modulation and high-quality factor. These data constitute important evidence for the gas fraction inside magmatic fluids. However, the mechanism behind this unique signal remains not fully understood. Here we report new laboratory evidence showing that two different processes have either scale-invariant or scale-dependent effects in generating tornillo-like events. These processes are respectively the gas pressure gradient, which triggers the event and regulates the slow decaying coda, and the fluid resonance into small scale structures which, in turn, control the frequency content of the signal. Considering that the gas pressure gradient is proportional to the fluid flow, these new findings, as applied to volcanoes, provide new information to better quantify both gas rate and volume, and the dimension of the resonator.

## Introduction

## Tornillo Seismic Events

Interpreting the diverse seismological signals generated by active volcanoes is fundamental in order to better understand the physics of the underlying process. Among these signals, the class of volcano-tectonic (VT) earthquakes are connected to the stability of the local volcanic system and thought to be diagnostic of deformation processes within it^1^, particularly of rock shear failure^2^. Conversely, seismo-volcanic signals resonating at lower frequencies, and hence known as Low Frequency Seismicity (LFS) events, are linked to fluid-movement processes^1^. The LFS class contains a number of sub-classes, with one particular type having a unique waveform: due to its screw-like shape, known as tornillo, Spanish for screw^3–5^. These signals have been observed on many different volcanoes around the world such as Galeras^4^, Puracé^6^, Asama-yama^7^, Tongariro^8^, Mt. Griggs^9^, Kelut^10^, Lokon Volcano^11^, and Vulcano^12^. Historically, LFS events were originally included in the Long Period (LP) signal sub-class due to their similar shallow location (up to 1.5 km depth), with effective monitoring only achieved by seismic stations near the active craters^6,8,10,12^. Tornillo’s recent classification as standalone class has evolved due to their characteristic waveform^12^ and their source mechanism^13^, due in part to more effective near-field instrumentation. They have been observed either as individual events or in swarms^5^ and in different regimes of volcanic activity, i.e. either pre-, co- and post-eruptive phases and in quiescence^5,6^. A good example of the significance of the tornillo class is that of Galeras (Colombia), where the choked volcanic conduit caused the pressurization of the volcanic system and the following explosive activity in 1992–1993. Tornillos were recorded before the explosive volcanic events and were linked to the steadily decreasing gas emissions as the eruption approached^14^. Tornillo-shaped infrasound events have also been recorded, most recently at Cotopaxi volcano (Ecuador) as described by Johnson *et al*.^15^.

**Figure 1:**
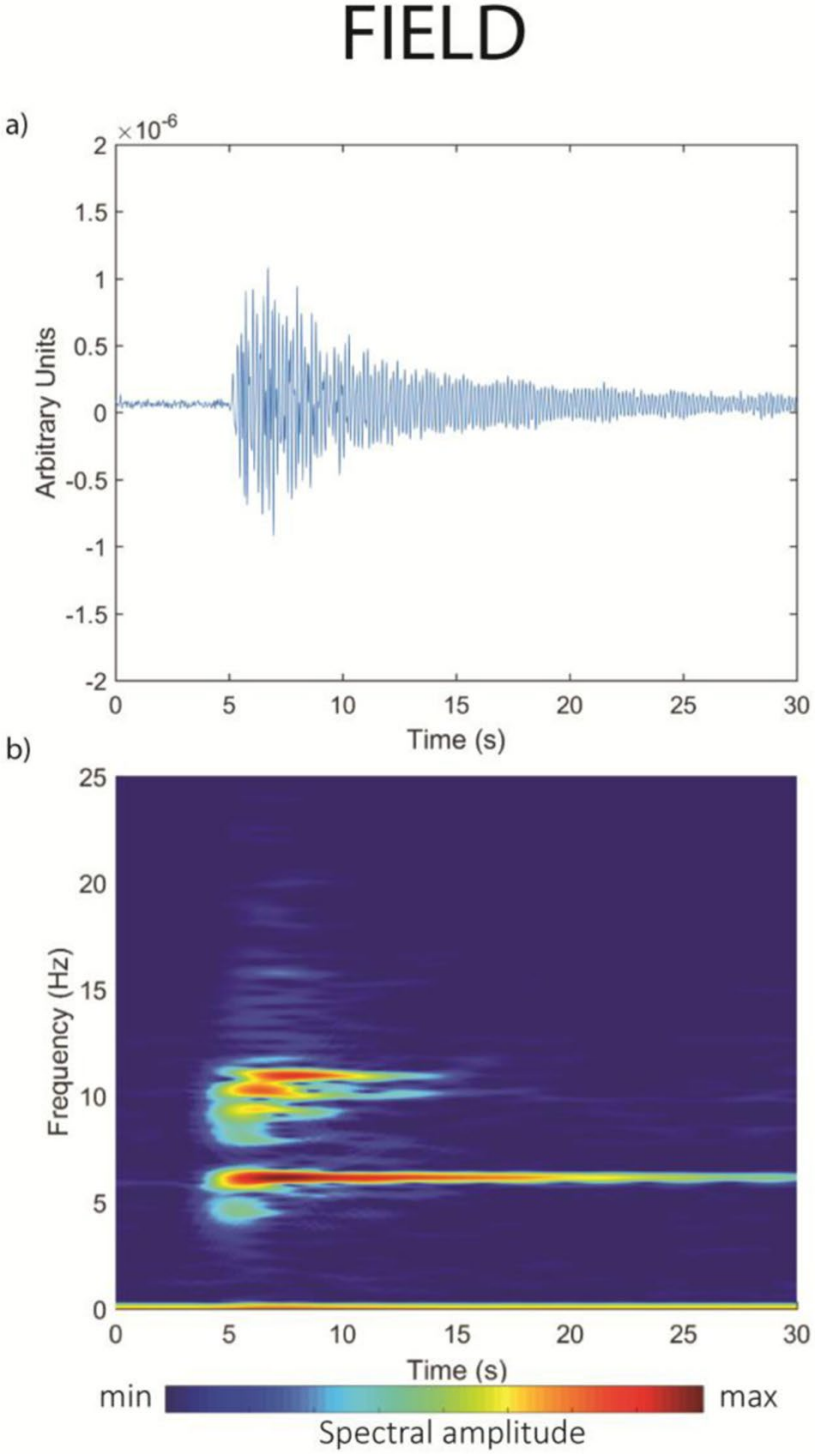
Field seismo-volcanic signal. (**a**) Waveform and (**b**) spectrogram of a tornillo event recorded on Vulcano Island (Italy) on November 7, 2008^12^. Modified from original publication^42^.

Tornillos are characterized by a combination of an impulsive/emergent excitation, a resonant vibrator with a characteristic amplitude modulation feature^12^ and an exponentially decaying Hilbert envelope^16^ causing a long (up to several minutes) quasi-linear slowing decaying coda^12^ (Fig. 1a). Their onset is emergent or impulsive, often with a positive first arrival^5^ and waveforms recorded at different sites have a high degree of similarity^17^. In terms of frequency content, tornillos have a quasi-monochromatic waveform or few (1–3) narrow spectral peaks (Fig. 1b), which are preserved at different seismic stations^4,5^, although some multichromatic events have been observed at Galeras^16^.

Similarly to LPs and volcanic tremor, the generation of tornillos was thought to be due to the resonance of a fluid-filled crack or a conduit in response to a pressure transient^12,18,19^, with the higher quality factor (Q) compared to LP signals attributed to water-filled cracks^10^, a higher gas fraction^2,20^, or the small aspect ratio cracks^21^. Other theories include the lumped-parameter model^3^ and a model based on the self-excitation of flow, yielding eddy shedding and turbulent (slug flow) oscillations^22^. However, none of these models could account for all available observations and incorporate tornillo characteristics^16^. Therefore, the appearance of tornillo events may be an indication of physical interaction (and conditions) between fluid flow and the surrounding conduit material. In particular, an increase in signal’s duration and a decrease in the dominant frequency preceding an eruption is frequently observed, likely due to an increase in gas fraction^4,5,10,12^. Tornillos at Tongariro have been localized just below the condensate layer, where a gas-filled region is known to be present^8^. However, a pure gas phase or gas-liquid mixture is not able to generate a sufficient impedance contrast between the fluid and the conduit (hence large Q) and instead a gas-ash mixture is considered as the oscillating fluid^8,20^. While several authors^2,17,20^ assigned a major role to the interaction between the gas fraction and the solid walls in generating these unique seismic events, this relationship was never quantified.

Therefore, to investigate and to better understand these fluid-induced signals, we have developed a laboratory setup enabling us to measure and control parameters such as temperature, axial and confining stresses, and pore pressure. We use Nitrogen gas as the key phase, deliberately venting the highly-pressurized gas stored within a borehole and a highly cracked region naturally formed from a shear/damage zone of basalt (Mt. Etna, Italy), rapidly to atmosphere.

## Methods

**Figure 2:**
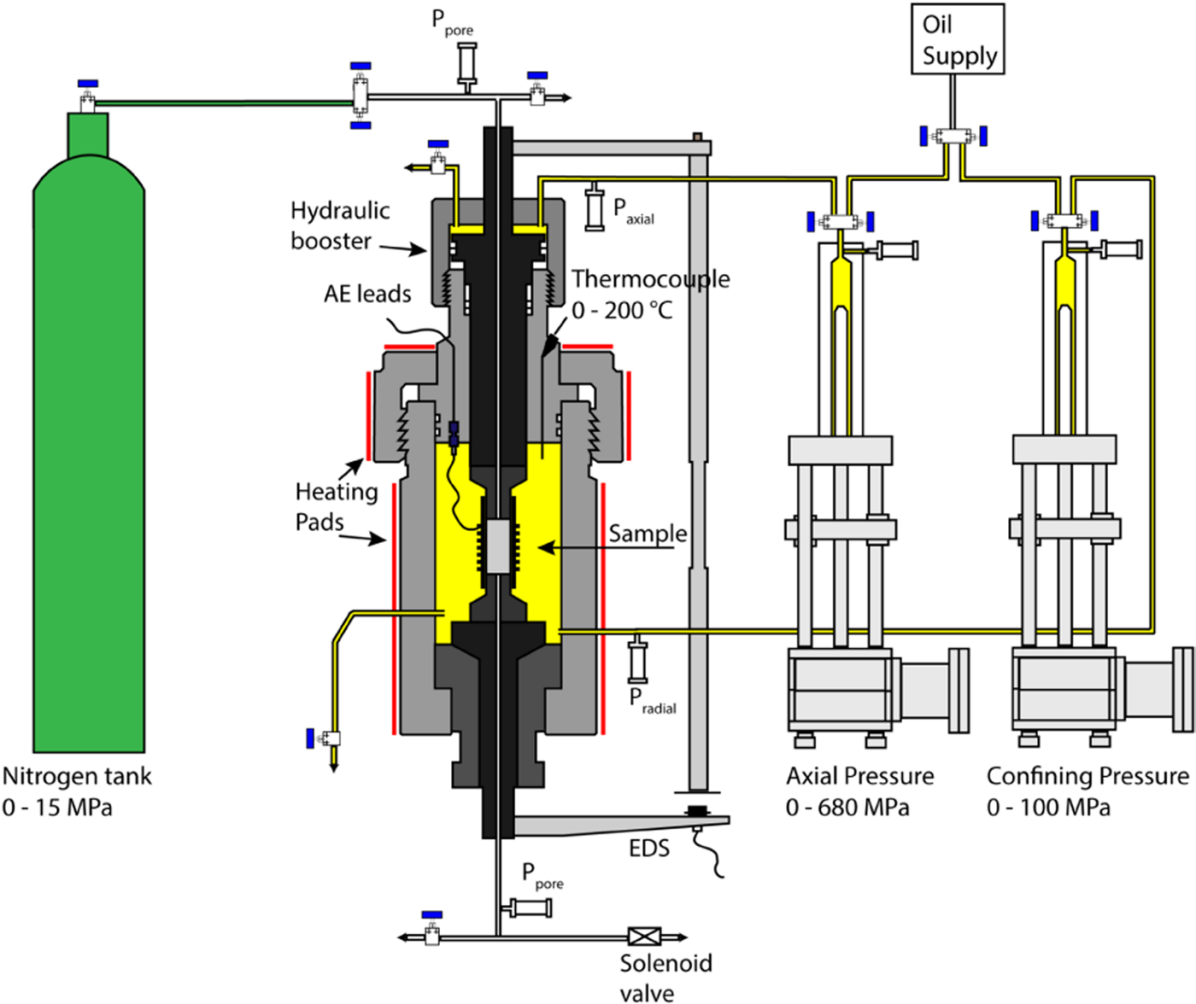
Schematic overview of the Sanchez triaxial apparatus. Modified from original publication^23^.

The rock used for this study was an alkali, porphyritic basalt collected from the southern flank of Mt. Etna (Italy). Basalt cylindrical samples (100 mm in length and 40 mm in diameter) were drilled with an axial conduit (3 mm in diameter) using a diamond core drill. Density and porosity were measured on 29 specimens (before being axially drilled), yielding a density of 2860 kg/m^3^ ± 10 and porosity of 2.05% ± 0.23. Triaxial deformation experiments were performed at the Rock Mechanics Laboratory at the University of Portsmouth (UK), using a conventional servo-controlled triaxial apparatus^23^ (Sanchez Technologies, Fig. 2) in dry conditions, with confining pressure (p_c_) of 30 MPa and temperature of 25 °C. Deformation was imposed at a constant strain rate of 10^−5^ s^−1^, up to and 1 minute beyond the dynamic failure. At this point the differential stress was removed to reach hydrostatic stress conditions and lock the newly created damage zone. Following this stage, nitrogen gas was injected inside the sample and pressurized up to 10 MPa. To maintain the same effective pressure of 30 MPa, the hydrostatic stress was brought to 40 MPa (corresponding to a depth of 1.4 km), at the same rate of the gas pressure. Once equilibrium was reached, gas pressure was released by a solenoid valve, placed beneath the lower end of the sample. The triaxial apparatus was equipped with external displacement and pressure transducers, connected to a high-speed data logger, in order to record the mechanical data at high sampling frequency (5 kHz). For the pore pressure, two transducers were used, connected to the upper and lower end of the sample respectively, with the latter being close to the solenoid valve. Microseismic events, known as Acoustic Emissions (AE) in a laboratory context, were recorded by 12 custom-made PZT transducers (Roditi 5A compressional crystals), with central frequency at 1 MHz, at a sampling frequency of 10 MHz. The transducers were installed in an engineered FKM-B rubber jacket^24^, in which the cylindrical sample was held. Output from the sensors was first band-pass filtered (40 kHz–1 MHz), to remove low frequency noise (<40 kHz) and high frequency electrical interferences (>1 MHz), and amplified with a gain of 30 dB. Using a bespoke AE data digitization system (ITASCA-Image) AE data (voltages) were recorded continuously around the time of pore pressure (p_p_) release and stopped once AE activity had ceased.

Complementing the laboratory dataset we present waveform data of a tornillo event recorded by the IVCR permanent seismic station on Vulcano Island on November 7, 2008^12^. This station is part of the Vulcano seismic network run by the Istituto Nazionale di Geofisica e Vulcanologia, Osservatorio Etneo - Sezione di Catania, Italy and composed of broad-band 3-component digital seismometers recording at a sampling rate of 100 Hz^12,17^. To compare with our 1-component (vertical) sensors, only the waveform recorded by the vertical component was processed.

To determine the relationship between pore pressure difference and fluid flow speed, we first apply Newton’s second law of motion and the pressure-gradient force equations.

The pressure difference between the two ends of the sample generates a pressure-gradient force, which considering the Newton’s second law of motion gives:

1 $$F=m\cdot a$$

where *F* is the force, *m* is the mass of a cubic fluid parcel of a fluid and *a* is the acceleration. The mass of the cubic parcel can be written in terms of the product between the density (*ρ*), the surface area (*dA*) and the height (*dz*):

2 $$m=\rho \cdot dz\cdot dA$$

The pressure-gradient force can now be written as the product of the pore pressure difference (*dP*) and the surface area (*dA*):

3 $$F=dP\cdot dA$$

Combining Eqs 2 and 3, Eq. 1 can be expressed as:

4 $$dP\cdot dA=\rho \cdot dz\cdot dA\cdot a$$

Rearranging Eq. 4:

5 $$a=\frac{dP}{\rho \cdot dz}$$

Equation. 5 shows that the acceleration of a parcel of fluid is directly proportional to the pressure difference across a surface. By integrating the acceleration over time, we now calculate the flow speed of the moving fluid, allowing a direct correlation to be made to the measured pressure difference.

## Results and Discussion

**Figure 3:**
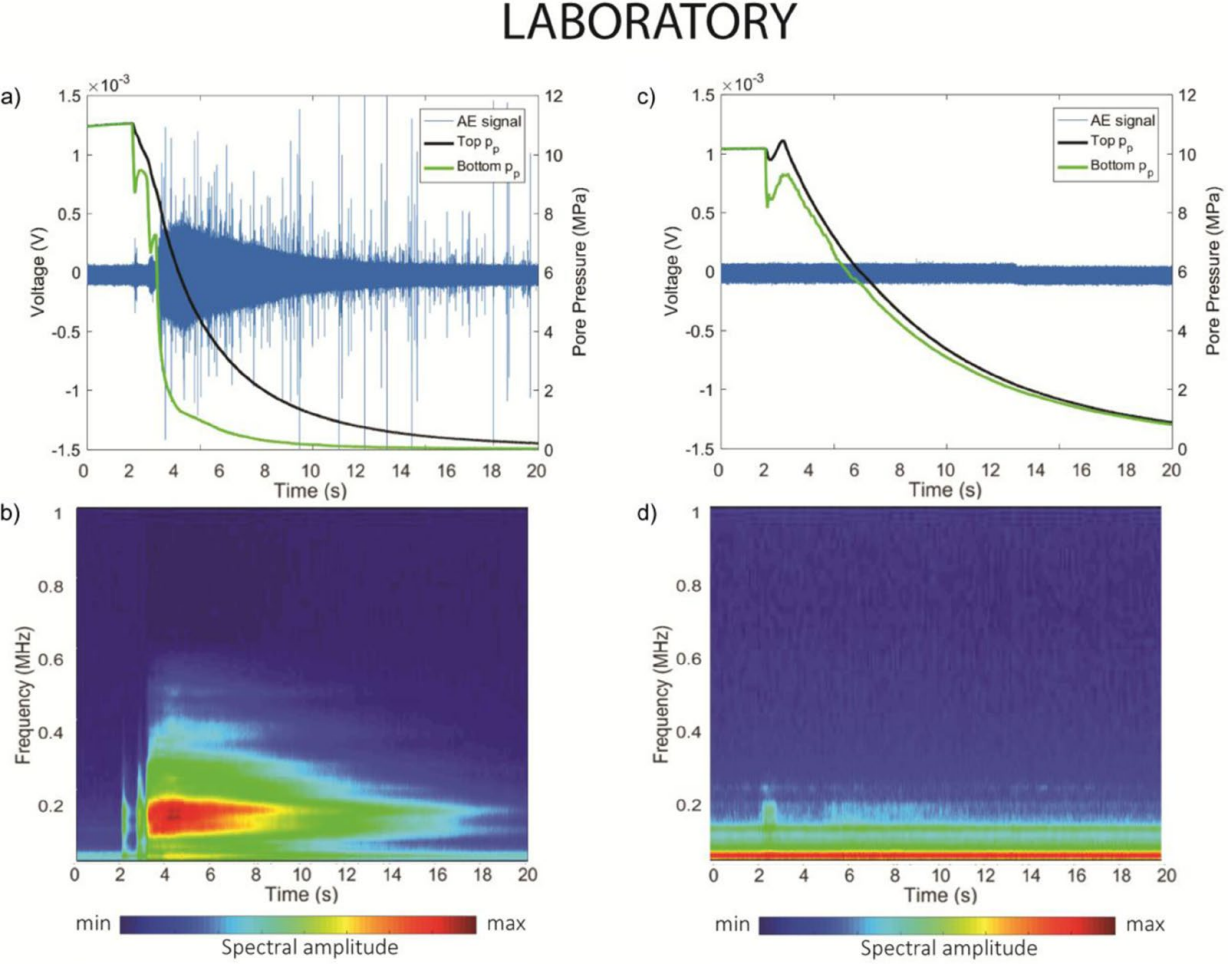
Laboratory AE signals. Waveform and spectrogram of the AEs, superimposed on the pore pressure curves, recorded during the release of pressurized Nitrogen gas in EB31 (**a**,**b**) and EB35 (**c**,**d**).

During pressure release in experiment EB31, a characteristic AE is recorded (Fig. 3a,b). The onset of this AE event occurs at the time of the gas pressure release (t = 2 s), generating two short-lived pulses before generating a screw-like shaped waveform lasting approximately 15 s. Each pulse was concurrent with a short-lived increase in pore pressure recorded at the bottom of the sample, while both top and bottom pore pressure continuously decreased (at different decaying rate) during the long-duration signal.

Qualitatively, there is a high degree of similarity between the continuous AE signal and that of a tornillo event. As an example, we report an event recorded at Vulcano Island, Italy^12^ (Fig. 1a,b). Both show durations of some tens of seconds, meaning that the AE signal has a remarkably long-duration (AE signals typically range from microseconds to milliseconds^25,26^), with a exponentially-decaying coda, giving both waveforms the characteristic screw-like tornillo shape. However, a clear difference between the two signals is also evident: while the waveform of the tornillo event appears to be a single event, the waveform of the AE event is characterized by the concurrent presence of numerous transients. These do not modify the overall shape of the continuous signal, and are likely explained in terms of sampling frequency (100 Hz in the field and 10 MHz in the laboratory), which allows short-duration events to be recorded in the laboratory experiment but are naturally filtered out in the field. Although the tornillo event (Fig. 1a) and its laboratory analogue (Fig. 3a) originated at very different scales, they are in general agreement when applied to a simple size (*d*) – frequency (*f*) scaling law^25,26^. And, although the resonating frequency is scale-dependent, the similar duration for both field and laboratory signals suggests a scale-invariant process behind the slowly decaying coda. We also note that AE signals lasting for tens of seconds has never been reported.

### Scale-invariant process

**Figure 4:**
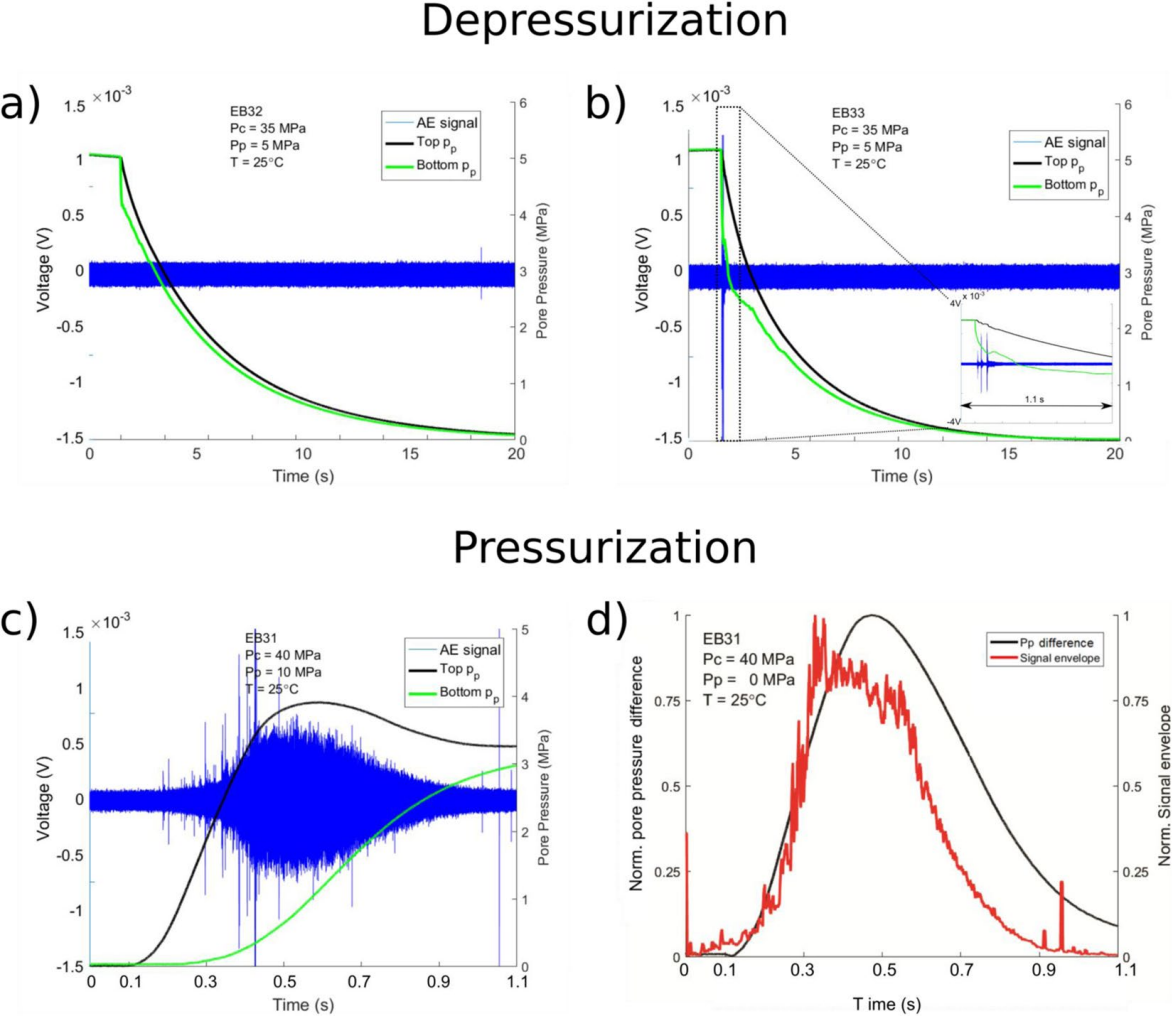
Importance of pore pressure difference. Waveform of the AEs, superimposed on the pore pressure curves, recorded during the depressurization of Nitrogen gas in EB32 (**a**) and EB33 (**b**) and during the pressurization stage in EB31 (**c**). The inset in (**b**) shows a 1.1s-long time-window with short AE activity in correspondence of increase pore pressure difference. (**d**) Normalized AE signal envelopes (red line) superimposed on the normalized pore pressure difference (black line) for the pressurization phase during experiment EB31.

To understand the key links between pore pressure, signal duration and the associated slowly decaying coda, comparison is made to a second experiment run at the same pressure conditions (experiment EB35, p_c_ = 40 MPa, p_p_ = 10 MPa, Fig. 3c,d). In this case no long-duration AE activity was recorded. However a short-lived signal, visible in the spectrogram around t = 2 s (Fig. 3d), occurs concurrently with a sudden decrease in the lower pore pressure, and not followed immediately by the top pore pressure. Within approximately 1 second, the signal weakens concurrently with both pore pressure curves, reaching similar values and decaying at similar rate. Again taking a simple qualitative comparison, we note that: (1), the gas pressure release is necessary but not sufficient to generate long-duration AE activity on its own and (2), a pore pressure difference is a fundamental requirement to generate long-duration AE activity. This trend was evident across 4 experiments run with Nitrogen (Figs 3 and 4a,b) and even during the pressurization stage (Fig. 4c). Here, also experiment EB32 (Fig. 4a) and EB33 (Fig. 4b) do not show enough pore pressure difference to generate a continuous signal, although a short-lived high difference is linked to transients signals in EB33 (Fig. 4b, see inset). Conversely during the pressurization stage in EB31 (Fig. 4c), one observes a faster increase of the top pore pressure evolution compared to the bottom pore pressure. This is then accompanied by a 1-second period where the waveform amplitude fades out as the pore pressure approaches equilibrium.

Similar behaviour was observed across 10 experiments with the release of either liquid water or superheated water due to steam forming as a result of the depressurization^27^. However, as reported in Fazio *et al*.^27^, the signals generated at elevated temperatures had duration in the order of tens of milliseconds in both scenarios, almost 1000 times shorter than the events reported here (Fig. 3a). Previous studies^14,20^ found that a longer signal is expected when dusty gases are involved, followed by mixtures of ash and SO_2_ and finally using mixtures of H_2_O and water droplets. In addition, by looking at the quality factor (Q) of LP events induced by the different fluid mixtures^20,21^, it is shown that ash-gases mixture are responsible for the highest Q (followed by H_2_O and water droplets) while bubbly water accounts for the lowest Q. Therefore we assume that the duration of the signal increases with decreasing liquid water content, in agreement with the results of this study and Fazio *et al*.^27^. Finally, we note that as the Nitrogen flows through the fractured rock, it is likely that the finest comminuted material is incorporated in the flowing fluid, hence forming a dusty gas.

Although the fluid type is important, it is clear that this is not sufficient on its own to generate long-lasting signals (e.g. in EB35, Fig. 3c,d). The same applies for the highest compressibility of the gas phase, which is responsible for slower pore pressure decay (tens of seconds), but not sufficient to cause sustained AE activity (Fig. 3).

Instead, the key to understand the long signal recorded in EB31 (Fig. 3a,b) is the pore pressure difference.

**Figure 5:**
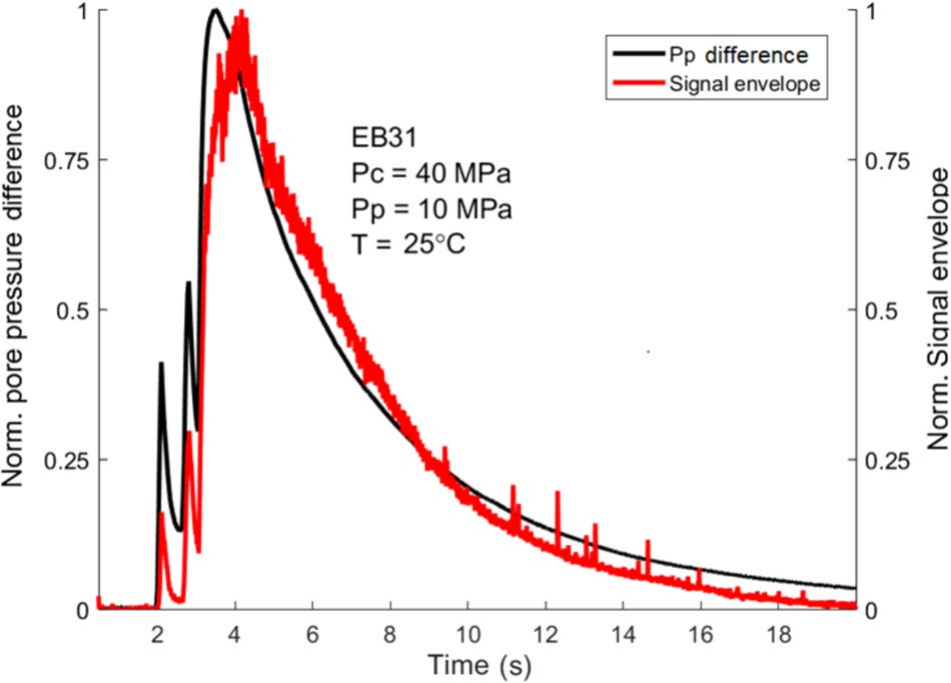
Signal envelope vs pore pressure difference. Normalized AE signal envelopes (red line) superimposed on the normalized pore pressure difference (black line) for the experiment EB31. The match between the two curves is proved by the high correlation coefficient, 0.94. Modified from original publication^42^.

Although a fully-quantitative comparison of the energy in these cases, compared to the field scenario, is beyond this paper, we have instead developed a semi-quantitative model to evaluate the relative importance of the pore pressure difference (Δ*P*, measured between the top and bottom end of the sample) on the characteristic long-duration waveform. This is achieved by taking the normalized AE signal envelope and the normalized Δ*P* over the entire 20-s-long window (Fig. 5). These data show an excellent agreement with a correlation coefficient of 0.94. Each spike in the normalized Δ*P* curve was picked by the signal envelope, as well as maintaining the proportion between them. The same analysis has been applied on the shorter signal acquired during the pressurization stage (Fig. 4d), also returning a correlation coefficient of 0.94. By using Newton’s second law of motion and pressure-gradient force equations (see Eq. 5) we then link the acceleration of a moving fluid to Δ*P* acting on the surface area of the fluid itself. Integrating the acceleration over time, we retrieve a proxy for fluid flow speed which is directly proportional to Δ*P*. In this way, the AE sensors are essentially acting as flowmeters, recording the flow speed of the fluid as the gas is vented out of the sample, with the decaying envelope of the signal mirroring the variation in fluid slow speed. Dinardo *et al*.^28^ came to a similar conclusion, finding linear relation between the flow rate of water pumped into a pipe and the vibration of the pipe wall itself.

The correlation between acoustic emission activity and flow rate is analogous to the relationship between the seismic eruption tremors and the magma discharge rate found by Ichihara^29^ in sub-Plinian eruptions at Shinmoe-dake volcano, Japan. Although this relationship is not fully explained by current models, Ichihara^29^ postulates that the source of tremor cannot lie in the atmosphere but rather at the fragmentation level within the conduit. Therefore free gas, subject to a differential pressure, is likely to be present in this scenario, similarly to our laboratory experiments. In particular, since our pressure conditions are equivalent to a depth of 1.4 km, close to the source of the deepest tornillo recorded on Vulcano, we generate analogue pressure differences which, in turn, are known to cause vibrations of similar duration. This approach led us to develop the hypothesis that the duration of a tornillo event does not depend on the size of the source, but it is rather a scale-invariant feature, which depends on the fluid flow rate.

### Scale-dependent process

**Figure 6:**
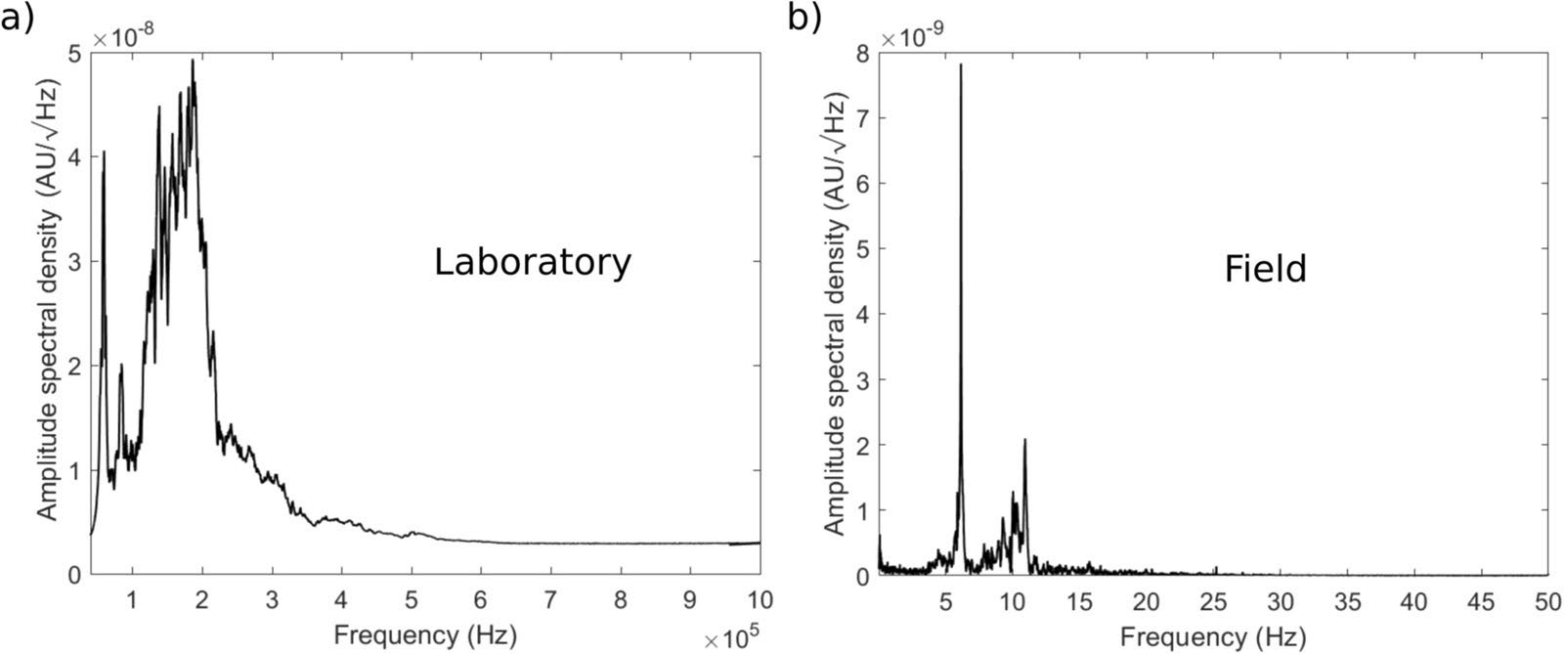
Lab vs field frequencies. Amplitude spectral density of (**a**) the laboratory AE signal (EB31) and (**b**) the field tornillo. In both cases two spectral peaks characterize the left side of the frequency spectra.

Analysing the frequency content of EB31 (Fig. 6a) (see methods), we observe that when sustained AE activity is generated two dominant frequency peaks (a narrow peak at 60 kHz and a broader peak between 100 and 200 kHz) characterize the spectrum. This is similar to the case of the tornillo event at Vulcano (Fig. 6b) where two spectral peaks (with a broad higher frequency peak and a narrower lower frequency peak) also characterize the spectrum.

**Figure 7:**
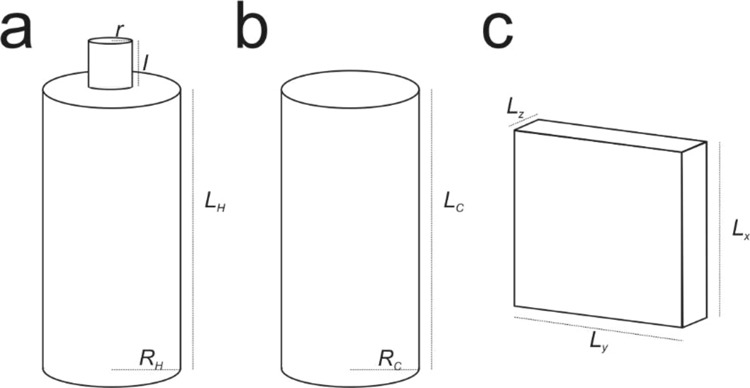
Sketch of three types of resonators: (**a**) Helmholtz, (**b**) tube/conduit, (**c**) rectangular cavity.

To understand the frequencies recorded at the laboratory scale, we model the source of the AE signal by taking into account three distinct resonance mechanisms of the sample fractures where the fluids move: Helmholtz, tube and rectangular cavity (Fig. 7).

The Helmholtz resonator has been widely used to model the source of infrasonic and seismic signals recorded in volcanic environments^30–33^, as well as for tornillo events^14,16^. This source mechanism allows wavelengths to be generated that much larger than the physical dimensions of the resonator used^ 31^. A Helmholtz resonator, consisting of a rigid walled cavity with a neck, reverberates with a single frequency^34^:

6 $${f}_{H}=\frac{c}{2\pi }\sqrt{\frac{S}{Vk^{\prime} }}$$

where: *c* is the sound speed, *S* is the area of the neck, *V* is the cavity volume, and *k*′ is the neck effective length calculated as *k*′ = *l* + 1.45 * *r* where *l* is the neck length and *r* is the neck radius (Fig. 7a). *V* was estimated as the volume of the cylinder of height *L*_*H*_ and radius *R*_*H*_. The sound velocity *c* of nitrogen at ~300° K is approximately 354 m/s. To test if the Helmholtz resonator can be responsible for the peak frequencies observed in the laboratory tornillo (60 kHz peak and a broadband 100 and 200 kHz), we systematically changed *l* and *r* within the ranges 0.1 mm–100 mm (the length of the axial conduit, i.e. the cavity) and 0.1–1.5 mm (the radius of the cavity), respectively. Both *L*_*H*_ and *R*_*H*_ were fixed to 100 − *l* (i.e. *l* + *L*_*H*_ = 100 mm) and 1.5 mm respectively. We also assume that during the release of the pressurized gas, the fluid flow along the 100-mm-long, 3-mm-wide axial conduit was impeded by rock particles as observed post-test due to the main fracture geometry^27^, forming the neck of the Helmholtz resonator.

**Figure 8:**
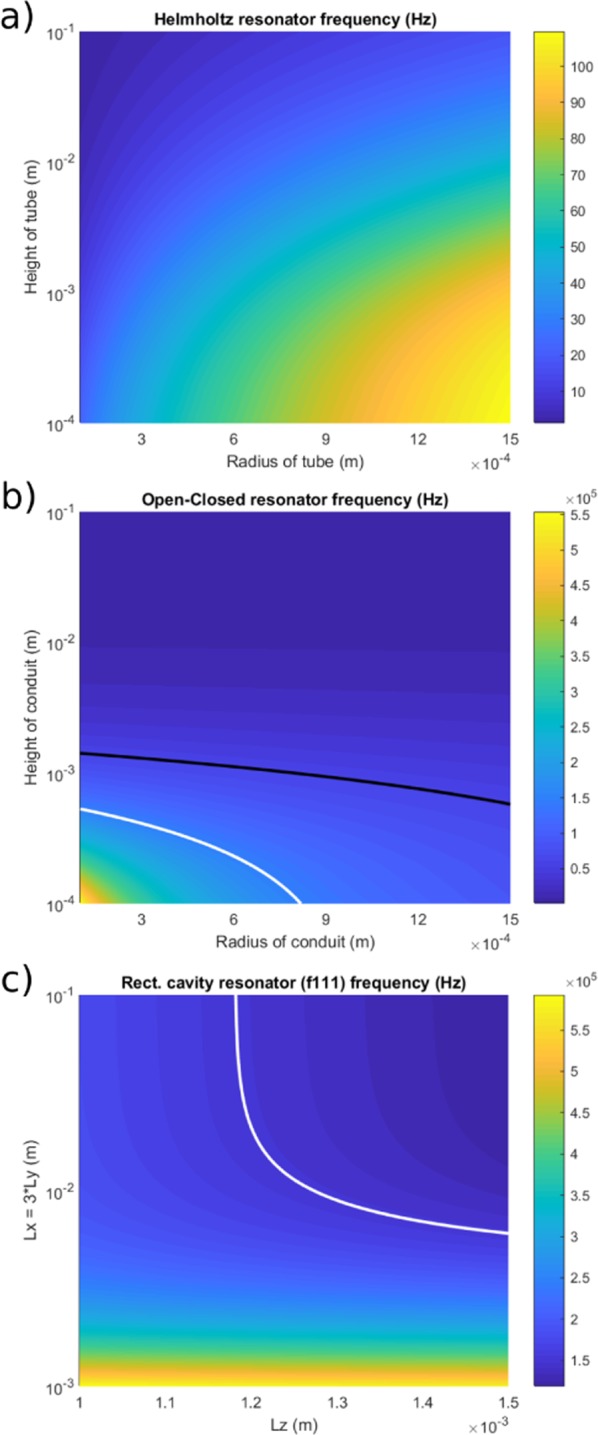
Simulation of the variation of theoretical frequency with three different resonator mechanisms. Variation of theoretical frequency for (**a**) the Helmholtz resonator, (**b**) the open-closed conduit (the black line and the white lines indicate the observed (experimental) frequency peak at 60 kHz and at 150 kHz respectively) and (**c**) the rectangular cavity (oblique mode, with the white line indicating the frequency peak at 150 kHz) as obtained by systematically changing the dimension parameters.

Using these boundary conditions, it is evident that the Helmholtz resonance frequency is too low to explain the observed peak frequencies of the laboratory tornillo (Fig. 8a).

This model has been used to explain the resonator at Galeras responsible for the generation of tornillos, with two crucial caveats: (1) a steady and slow gas accumulation and (2) a fluid pressure above a certain threshold^14^. However neither are applicable for our laboratory-analogue tornillo events. In fact gas pressure discharge (or accumulation for the pressurization stage of EB31, Fig. 4c) was not steady but decelerating in all cases (Figs 3a,c and 4a,b), the same fluid pressure was present in EB31 and EB35 (Fig. 3a,c respectively) and only in EB31 was a tornillo-like event generated. Therefore, in addition to the frequency analysis, the Helmholtz resonator cannot explain our longer-lasting AE signals.

A tube or conduit, considered as a 1D resonant source (Fig. 7b), has been used by several authors to model the generation mechanisms of volcano seismic and acoustic signals^35–39^. In the conduit-resonator case, the resonating frequencies depend on the boundary conditions. In particular, a conduit with both open ends reverberates with frequencies:

7 $${f}_{T1}=\frac{nc}{2({L}_{C}+\delta L)}$$

where *n* is the mode index (*n* = 1, 2, …), *L*_*c*_ is the length of the conduit and *δL* is the open end correction, approximately equal to 0.6 times the conduit radius *R*_*c*_. Focusing on the fundamental mode, *n* was fixed to unity. Considering the 100 mm-long, 3 mm-wide axial conduit as an open-open tube, this would resonate at 1746 Hz, lower than the observed frequencies. However, if we assume that the conduit had a closed end (similarly to that assumed for the Helmholtz resonator), we calculate:

8 $${f}_{T0}=\frac{(2n+1)c}{4({L}_{C}+\delta L)}$$

where *n* is the mode index (*n* = 0, 1, 2, …), which was fixed to 0 to represent the fundamental mode. *L*_*c*_ and *R*_*c*_ are set to the ranges 0.1–100 mm and 0.1–1.5 mm, respectively. It is now evident that both frequency peaks measured in the experiment can be generated, assuming open-closed cylindrical cavities with sub-millimetric dimensions (Fig. 8b).

The last resonance mechanism option is the resonating rectangular cavity, or fluid-filled crack, commonly used to explain all types of LFS^2^, but also tornillo specifically^10,40^ and infrasonic harmonic tremor^31^. The resonance frequencies within the cavity are defined here as^31^:

9 $${f}_{lmn}=\frac{c}{2}\sqrt{{(\frac{l}{{L}_{x}})}^{2}+{(\frac{m}{{L}_{y}})}^{2}+{(\frac{n}{{L}_{z}})}^{2}}$$

where *l*, *m*, *n* are the mode indices (*l*, *m*, *n* = 0, 1, 2, …), *L*_*x*_, *L*_*y*_, and *L*_*z*_ are the dimensions of the rectangular cavity (Fig. 7c). The standing waves in a rectangular cavity can be divided into three categories^41^: (i) axial waves, characterized by two mode indices equal to zero; (ii) tangential waves, characterized by a mode index equal to zero; (iii) oblique waves, characterized by all indices nonzero. The axial modes have higher amplitude than the tangential modes, that are more energetic than the oblique modes^31,41^. Assuming *L*_*x*_ = 3 * *L*_*y*_^42^, we systematically changed *L*_*x*_ (and consequently *L*_*y*_) in the range 0.1–100 mm and *L*_*z*_ in the range 1–1.5 mm. The range in values of *L*_*z*_ represents the damage zone maximum width, as confirmed via a thin section of the post-test sample, considering the further aperture due to the pressurized gas. Focusing on the fundamental oblique mode *f*_111_, only the peak frequency at 150 kHz, as measured in the experiment, is consistent with standing waves in a millimetric size cavity (Fig. 8c). While the frequency peak at 60 kHz can only be explained by the vibration of an open-closed conduit (or a segment of it), both the rectangular cavity and the open-closed conduit can justify the frequency peak at 150 kHz. However, we rule out the rectangular cavity source model for the following reasons: firstly, although we observed fracture aperture up to 1.5 mm, this happened for a very short portion of the fracture (1–2 mm), with the majority of the fracture showing aperture less than 1 mm; secondly, even considering the smallest possible aperture responsible for the 150 kHz frequency peak, the cavity should be 10 mm long. The observed fracture appeared highly irregular over such length, therefore not being represented by a rectangular cavity. In addition a high amount of comminuted particles (with particles long up to 0.5 mm) were found within the fracture. Instead, the axial conduit, which originally had smooth surfaces, contained fewer particles and maintained smooth surfaces even after the formation of the damage zone.

Taking these observations and results together, it is likely that the source of tornillo-like events in our experiments is not the vibration of the rectangular cavity representing the fracture zone but instead multiple open-closed sections within the centrally pre-drilled conduit, which cannot be modelled as a Helmholtz resonator.

Due to the protocol used, these open-closed sections of the conduit were formed after the formation of damage zone, during the triaxial deformation phase. Although it appears that the shear fracture is not directly responsible in generating the tornillo-like event, the deformation and the failure of the sample provided the material to choke the conduit (closed end) or to obstruct the flow of gas through it (open end). This occurred during experiment EB31, allowing the build-up of pressure difference and in turn the generation of tornillo-like events. Conversely, during experiment EB35 the conduit was relatively free, no significant pressure difference was observed, and no long-duration AE signal. The role of the fracture is also confirmed by previous data^23^, showing that the presence of the conduit alone is not sufficient to generate long-lasting signals. These results explain the occurrence of tornillos with multiple spectral peaks, which are explained in terms of vibration of multiple proximal open-closed conduits subject to a critical flow speed of a gas-rich phase.

Our model of an open-closed conduit characterized by a critical flow rate of magmatic fluids is in partial agreement with the observations at Galeras. Although we have identified a resonator mechanism different from the Helmholtz resonator and different pressurization rate, key roles in generating tornillos are played by a choking conduit and a high magmatic pressure gradient, which in turn generates a critical flow rate. We may further speculate that the increased signal duration, followed by a decrease just before eruption^27^ may indicate an increased flow rate due to a higher pressure gradient within the conduit, followed by the breech in the obstructed conduit, leading to a temporary release of the internal pressure before the eruption commences. Therefore, our findings can be applied in understanding magmatic pressure gradients and flow rate within open-closed conduit as tornillo events are observed and this information can be used to understand whether the eruption is explosive and the rate at which magmatic fluids are ejected.

As previously demonstrated^3^, flow speed is of critical importance in triggering a sustained seismic response, supported experimentally by our laboratory data: long-duration microseismicity occurs only when the flow speed exceeds a critical threshold. In addition we proved here that a critical flow speed, theorized for laminar fluid^3^, is also required for non-laminar fluid.

## Conclusions

In this study, gas depressurization experiments, through a fractured sample, were performed in order to study the fluid-induced AE activity and to compare such activity to analogue field volcano-seismic events. In the presence of a relatively high and lasting pore pressure gradient between the two ends of the sample, a sustained AE signal was recorded, with a waveform similar to the tornillo events found in several volcano settings. The duration of the laboratory signal is similar to that of its volcanic analogue, with the AE signal’s envelope mirroring the pore pressure difference behaviour, which in turn is proportional to the fluid flow speed.

Considering that the laboratory experiments simulate realistic pressure conditions, it can be assumed that field flow speed were successfully reproduced in the laboratory environment and that, therefore, this is a scale-invariant process. The frequency contents of the AE signal and the tornillo events are scale-dependent. On the basis of the theoretical fundamental frequencies computed for three representative models, Helmholtz, tube and rectangular cavity, and by assuming reasonable dimensions for the reverberating fractures, the only model we find that is capable of reproducing the measured AE signal peak frequencies is that of the open-closed conduit model.

This study provides new insight and understanding of the source of volcanic fluid-induced seismicity and has potential implications for volcano monitoring. Frequency domain analysis of tornillo waveforms data provide new information relating the shape and dimensions of the resonating crack while time-domain analysis reveals the gas pressure gradient within the volcanic edifice and the gas flow rate. These are parameters particularly important to characterize the volcanic gas budget, the deep seated volcanic plumbing system and ultimately the type and style of possible volcanic eruption. Future works are stile required, particularly the use of calibrated AE sensors and accurate measurement of the fluid flow, so as to better estimate the minimum gas volume discharged and how this links to the characteristics of recorded volcanic seismicity on the field.
